# Forgotten forests - issues and prospects in biome mapping using Seasonally Dry Tropical Forests as a case study

**DOI:** 10.1186/1472-6785-11-27

**Published:** 2011-11-24

**Authors:** Tiina Särkinen, João RV Iganci, Reynaldo Linares-Palomino, Marcelo F Simon, Darién E Prado

**Affiliations:** 1 Department of Botany, Natural History Museum, Cromwell Road, London SW7 5BD, UK; 2 Universidade Federal do Rio Grande do Sul, Programa de Pós-Graduação em Botânica, Av. Bento Gonçalves, 9500 - Prédio 43433, Bloco 4 - Sala 214, Campus do Vale, Porto Alegre- RS 91501-970, Brazil; 3 Herbario Forestal MOL, Universidad Nacional Agraria La Molina, Apartado 456, Lima 1, Peru; 4 Embrapa Recursos Genéticos e Biotechnologia, PqEB, Caixa Postal 02372, Brasilia-DF 70770-917, Brazil; 5 Facultad de Ciencias Agrarias, Universidad Nacional de Rosario. P.O. Box N°14, S2125ZAA Zavalla, Argentina

## Abstract

**Background:**

South America is one of the most species diverse continents in the world. Within South America diversity is not distributed evenly at both local and continental scales and this has led to the recognition of various areas with unique species assemblages. Several schemes currently exist which divide the continental-level diversity into large species assemblages referred to as biomes. Here we review five currently available biome maps for South America, including the WWF Ecoregions, the Americas basemap, the Land Cover Map of South America, Morrone's Biogeographic regions of Latin America, and the Ecological Systems Map. The comparison is performed through a case study on the Seasonally Dry Tropical Forest (SDTF) biome using herbarium data of habitat specialist species.

**Results:**

Current biome maps of South America perform poorly in depicting SDTF distribution. The poor performance of the maps can be attributed to two main factors: (1) poor spatial resolution, and (2) poor biome delimitation. Poor spatial resolution strongly limits the use of some of the maps in GIS applications, especially for areas with heterogeneous landscape such as the Andes. Whilst the Land Cover Map did not suffer from poor spatial resolution, it showed poor delimitation of biomes. The results highlight that delimiting structurally heterogeneous vegetation is difficult based on remote sensed data alone. A new refined working map of South American SDTF biome is proposed, derived using the Biome Distribution Modelling (BDM) approach where georeferenced herbarium data is used in conjunction with bioclimatic data.

**Conclusions:**

Georeferenced specimen data play potentially an important role in biome mapping. Our study shows that herbarium data could be used as a way of ground-truthing biome maps *in silico*. The results also illustrate that herbarium data can be used to model vegetation maps through predictive modelling. The BDM approach is a promising new method in biome mapping, and could be particularly useful for mapping poorly known, fragmented, or degraded vegetation. We wish to highlight that biome delimitation is not an exact science, and that transparency is needed on how biomes are used as study units in macroevolutionary and ecological research.

## Background

South America is one of the world's most diverse continents, housing around 90,000-110,000 species of seed plants, c. 37% of the world's total [1-3]. Taxonomic diversity, however, is not evenly distributed within the continent; on a broad scale, the Amazon rain forest is home to completely different species to those from the mountain tops of the Andes, and areas differ on a finer scale in their species richness and endemism [4]. Understanding such diversity gradients, and the processes that shape and maintain them, remains a focal question in ecology and evolutionary biology.

Studies aiming to understand diversity gradients rely on schemes depicting the distribution of this diversity. At the continental scale, species diversity is divided into major units referred to as biomes (also know as vegetation zones, phytogeographic regions, phytochoria, etc). For example, Africa is divided into 22 biomes based on floristic similarity, climatic factors, and vegetation structure. The African biome map was originally developed by White [5,6] and later revised and digitised using remote sensing data [7]. White's biome delimitation has been widely accepted among ecologists, conservationists and evolutionary biologists, and the stability of the African biome map has enhanced collaboration across research fields (e.g. [8-10]).

South America is the focus of much biodiversity research (e.g. [11-15]). In spite of this, a stable biome scheme comparable to that available for Africa is still lacking. Various digital biome maps are currently available (e.g. [16,17]), but these maps differ greatly in both how they depict diversity across the continent and in their principles of construction. The maps preferred by many ecologists are based on remote sensing data, where biomes are delimited based on vegetation structure (e.g. land cover map of South America [17]) (Table 1). Although remote sensing maps are superior in the fine detail they provide at regional and local level across the continent, such 'structural maps' do not consider floristic similarity and hence may not represent biologically meaningful units. In contrast, 'floristic maps' (i.e. maps which differentiate units mainly by their plant assemblages, but may also use biological and physical aspects) commonly used in biogeography, conservation biology and macroevolution are based on data on species composition and endemism, but represent stylised areas and lack detail at regional and local level (e.g. WWF Ecoregion map [16]). The poor spatial resolution of these 'floristic maps' potentially hinders their use for hypothesis testing in macroevolution and ecology. Ultimately, an optimal map would have greater spatial detail *and *include floristic data (Table 1).

**Table 1:** General types of biome maps

Map types	Baseline data	Spatial detail	Hierarchical representation of regions	Representation of ecological affinities	Representation of evolutionary communities	Examples of maps	End users
Floristic maps	Species composition, richness & endemism	Crude	Good	Poor	Good	WWF Ecoregions [16]	Evolutionary biology & conservation science
Land cover maps	Remote sensing data	Fine	Poor	Good	Poor	Land Cover Map of South America [17]	Ecology
Hybrid maps	Climate, elevation, and species composition	Fine	Good	Good	Good	Ecological Systems Map [31]	All research fields

Main types of biome maps summarised and compared.

The gap between the fields of ecology and evolution is closing (e.g. [18,19]). As a result, there is a growing need for a common frame of reference with which to test hypotheses that bridge the fields. Currently, biomes are used as study units in many macroevolutionary and ecological studies (e.g. [20-24]), without critical analysis of the biome maps used and their limitations. It is clear that a thorough discussion is needed on what biomes are and how they should be delimited. Defining a stable biome map scheme for South America that could be used in both macroevolutionary *and *macroecological research would increase transparency and stimulate dialogue between the research fields.

The concept of biomes was originally developed by Alexander von Humboldt [25] who first noted the dynamic relationship between vegetation composition and structure, climate, and geography. Humboldt argued that vegetation had a central role in the understanding of landscape level processes, and first articulated the idea that biomes can be seen as evolutionary theatres for the lineages they contain. The modern development of Humboldt's original concept, where biomes are seen as biological meta-communities, comes largely from recent molecular phylogenetic studies in which a strong pattern of phylogenetic niche conservatism (PNC) is seen across plant lineages at global level (e.g. [20]). The expectation that related species tend to occupy similar environments [26,27] is the basis for PNC. The PNC concept potentially has a strong role in explaining historical species assembly, as it governs the composition of regional floras and species pools from which communities are assembled over time [27]. This means that biomes, and their dynamic history through time, can have a strong effect on the evolution of the lineages they host (e.g. [14,28]).

This study was initially started with the aim of exploring ways to derive better biome maps for South America that could be used in biological research. In this paper we focus on exploring how herbarium data can be used as aid in biome mapping. First, we review a set of available digital biome maps of South America to discuss their strengths and weaknesses. We focus on five recently proposed biome maps which are available in digital format, including the Land Cover Map (**LCM**) [17], WWF Ecoregion map (**ECO**) [16], the Americas Basemap (**AB**) [29], Latin American Biogeography Scheme (**LAB**) [30], and the Ecological Systems Map (**ESM**) [31]. The comparison is performed through a case study of the Seasonally Dry Tropical Forests (SDTF), a relatively poorly known biome with a strongly fragmented distribution across South America. Georeferenced herbarium records of species endemic to the biome are used to ground-truth the biome maps *in silico*. In the second part of the study we propose Biome Distribution Modelling approach (BDM) to biome mapping. Climatic and elevation data is used in conjunction with herbarium specimen data of habitat specialist species to derive a new high resolution biome map for SDTF in South America based on predictive modelling.

### Seasonally Dry Tropical Forests

The SDTF, or BTES (Bosques Tropicales Estacionalmente Secos) or FED/FES (Florestas Estacionais Deciduais e Semideciduais), is a relatively recently identified biome, which was first defined based on floristic similarity and high endemism at both generic and species level [32,33]. The ecology and biology of neotropical SDTF have been recently reviewed [34-36], but in short, SDTFs are found in areas with low annual rain fall less than 1,100 mm/year with a dry season at least 5-6 months long during which rain fall remains below 100 mm [37,38]. The flora is dominated by species in the angiosperm families Leguminosae, Cactaceae, and Bignoniaceae, and species show morphological adaptations to the long dry season during which most of them are deciduous [38].

One of the reasons the SDTF biome has remained poorly understood is the high structural variability of SDTF vegetation. As defined by Prado [33] and Pennington et al. [34], the SDTF biome includes vegetation of widely differing structure from closed canopy forests to open scrublands. This structural variation has led to confusion between SDTFs and the other South American dry biomes, the savannas (e.g. Brazilian Cerrado) and the Chaco [39]. Whilst SDTF grow on rich soils and have a succulent rich flora that lack fire adaptations (e.g. Cactaceae), most savannas are dominated by grasses, occur on poor, aluminium-rich soils (e.g. the Cerrados [40]), and experience regular fires (Table 2). The Chaco, which is a temperate biome, differs from SDTF in experiencing regular frosts (Table 2) among several other environmental factors (see [41,42]).

**Table 2:** Definitions of South American dry biomes

Biome	Annual rainfall (mm/year)	Length of dry season (months)	Dominant plant families	Physiognomy of vegetation	Notes on flora	Soils	Natural fire cycles	Frost
Seasonally dry tropical forests [45]	< 1,100	5-9	Leguminosae, Bignoniaceae, Euphorbiaceae, Cactaceae, Bromeliaceae	Open to closed canopy forest	Adaptations to drought, scarcity of perennial grasses	Fertile, well drained, shallow soils. pH 6-7	Absent	Absent
Savannas [40,68,69]	800-2,200	3-5	Leguminosae, Myrtaceae, Vochysiaceae, Poaceae, Cyperaceae	Open to wooded grasslands	Fire adaptations in most plants, dominance of C4 grasses	Poor, Al rich, well drained, deep soils. pH very acid (~5)	Regular	Absent
Chaco [41,42]	450-1,200	c. 5 (variable)	Leguminosae (esp. Mimosoideae), Anacardiaceae, Cactaceae, Poaceae, Bromeliaceae	Open to closed canopy forest, interspersed with occasional savannas	Frost and salinity tolerant species with temperate affinities	Saline. Sometimes very alkaline in depth (up to pH 8-9)	Occasional	Regular, rarely snow

Comparison of the tropical dry biomes of South America and their definitions. See Werneck et al. [36] for a review on South American dry biomes.

**Figure 1:**
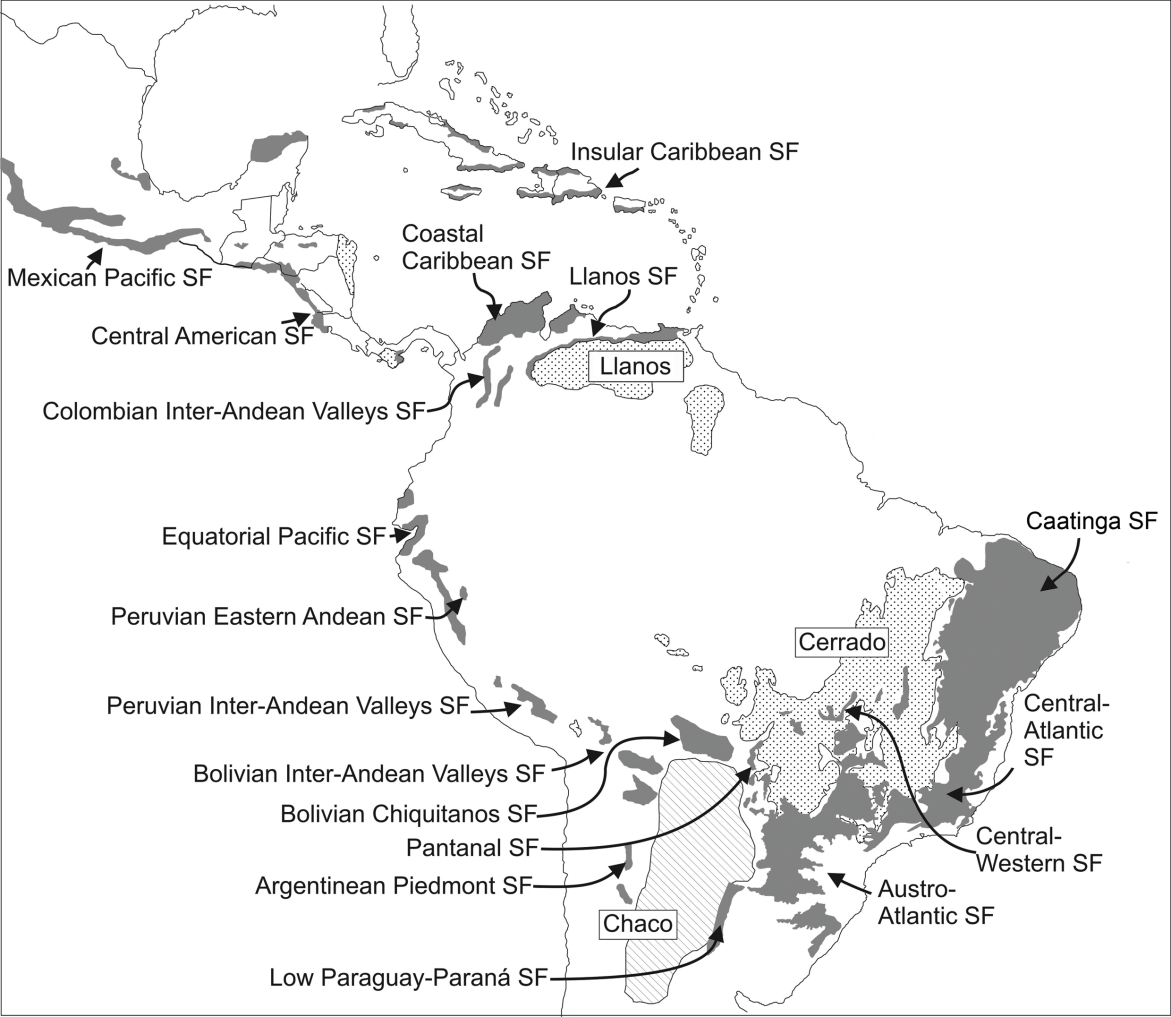
**South American dry biomes**. Distribution of the Seasonally Dry Tropical Forest (SDTF) biome in South America. The 18 major forest nuclei are labelled. The Caatinga nucleus as defined here includes North-East Brejo and Peri-Caatinga nuclei. The two other dry biomes, the Chaco and savannas (Llanos and Cerrado) are also shown for contrast and comparison. Figure from Linares-Palomino et al. [44].

The current distribution of SDTF in South America is highly fragmented due to both natural factors (e.g. climatic factors and fluctuations) and human disturbance [36,43]. Remaining forest areas have been divided into 18 isolated forest nuclei (Figure 1) [44]. The largest nuclei are found in north-eastern Brazil (Caatinga), in the Paraguay-Paraná river systems (Misiones), and in south-western Bolivia and north-western Argentina (Piedmont) (Figure 1) [45]. Two smaller but significant SDTF nuclei include the Bolivian Chiquitanía, and the coastal SDTF in northern Colombia and Venezuela (Figure 1). The least well characterized are the smallest fragments of SDTF found in the Andes along the inter-Andean valleys and the Pacific coast of Ecuador and Peru (Figure 1) [46]. Although the SDTF biome as a whole is generally poorly represented in biome maps, and is often confused with savannas and the Chaco, the Andean SDTF fragments are the most neglected and underrepresented due to their small size.

The fragmented distribution and the structural variation of the vegetation make the SDTF biome a perfect case study for exploring how herbarium data could be used as an aid in biome mapping. Firstly, fragmented biomes are generally underrepresented in biome maps as small areas often remain undetected in continental scale maps especially if spatial resolution is poor. The issue of how best to map small but significant biome fragments has not thus far been discussed in detail, although the need for this has been highlighted by conservation agencies [e.g. [16]]. Similarly, biomes that show structural variation propose a challenge due to the fact that many remote sensed applications cannot readily pick up on the differences between structurally similar vegetation. Validation methods such as ground-truthing are required for remote sensing maps, but solutions for continental scale studies (e.g. biome mapping) are sparse.

## Results

### Delimitation of SDTF on Available Biome Maps

The comparison shows large differences in how current biome maps depict SDTF biome distribution (Table 3). Maps based on species composition (AB, ECO, and ESM) perform best despite their poor spatial resolution (Tables 3 and 4). The WWF Ecoregion map is the best performing map showing 46.6% of specimens falling under SDTF (Table 3). There is a consistent pattern across the biome maps where large fractions of specimens fall into other dry biomes, 5.2-15.4% under Cerrado and 2.2-8.4% under areas labelled as Chaco (Table 3; raw results in Additional file 1, Tables S1, S2, S3, S4, and S5). Some areas labelled as Cerrado or Chaco receive > 20 hits SDTF habitat specialist species indicating that SDTF fragments exist within these biomes: ECO map includes 19, LCM six, and AB 13 of such areas (Additional file 1, Table S6).

**Table 3:** Performance of biome maps for SDTF

	Percentage of specimens					
Biome map	SDTF		Cerrado		Chaco	
	All species	Narrow endemics only	All species	Narrow endemics only	All species	Narrow endemics only
Latin American Biogeography Scheme (**LAB**) [30]	40.6	36.3	7.6	4.3	8.4	5.7
Americas Basemap (**AB**) [29]	42.7	42.0	29.9	15.4	3.9	3.1
WWF Ecoregions (**ECO**) [16]	46.6	43.6	9.9	5.2	5.7	4.9
Land Cover Map (**LCM**) [17]	14.6 (62.7^1^)	16.2 (57.3^1^)	6.3	4.5	2.2	2.2
Ecological Systems Map (**ESM**) [31]	36.4	32.4	13.9	6.6	6.7	5.5

Percentage of specimens which fell into the SDTF biome compared to the two other South American dry biomes, savannas (mainly Cerrado in our analysis) and the Chaco.

^1 ^Most specimens fall into agricultural land, and if these areas are considered as degraded SDTF, 62.7% of specimens fall into SDTF. This is likely an overestimation as part of the agricultural land could be degraded savannas or Chaco.

**Table 4:** Biome maps of South America

Biome map	Source publication	Primary data used	1^st ^tier		2^nd ^tier		3^rd ^tier		4^th ^tier	
			Label	No. of classes	Label	No. of classes	Label	No. of classes	Label	No. of classes
Latin American Biogeography Scheme **(LAB)**	[30]	Geography, and secondarily species composition and endemism	Dominions	2	Regions	3	Subregions	8	Provinces	55
Americas Basemap **(AB)**	[29]	Species composition and endemism	Biomes	6	Vegetation zones	22	Vegetation types	73	polygons	597
WWF Ecoregions **(ECO)**	[16]	Species endemism, but landform used as primary data in areas lacking widely used biogeographic maps e.g. South America	Biomes	11^1^	-	-	Ecoregions	117	polygons	3,311
Land cover map **(LCM)**	[17]	Remote sensing and elevation	Biomes	8	-	-	Land cover classes	65	polygons	71,153
Ecological Systems Map **(ESM)**	[31]	Climate, elevation, geology, land cover, and landform data	Domains	3	Divisions	22	Ecological systems	604	polygons	285,000^2^

Comparison of the available biome maps for South America. The hierarchical division of each biome map is shown, followed by the number of headings under each division. The number of headings includes areas within South America only, although some schemes extend to Central and North America. Human made habitats (e.g. urban areas, agricultural land) and barren areas (e.g. water, ice, and snow) were excluded from the comparison where possible. Tiers above continental scale (e.g. realms) are not shown.

^1 ^Total number of terrestrial biomes in WWF Ecoregion map is 14 but some of the major biomes not found within South America.

^2 ^Shape layers for the individual ecological systems are not available yet for the whole of South America. The number of polygons given is the current estimate. The currently available map is divided into 604 polygons.

Species-by-species breakdown of the results shows that there is a consistent trend across species where similar percentage of specimens fall within and outside SDTF (Additional file 2, Tables S7 and S8). Consistent percentage of specimens fall in either neighbouring biomes or other dry biomes (Additional file 2, Tables S7 and S8). This indicates that the results of the map comparison are not due to single species dominating the dataset, but due to a consistent trend across species. Similarly, analysis of the smaller data set where only narrowly restricted species were included shows a consistent pattern with the wider analysis, where less than half of specimens fall in SDTF (Table 3). The secondary analysis shows slightly smaller fractions of specimens mapping under Cerrado and Chaco (Table 3).

**Figure 2:**
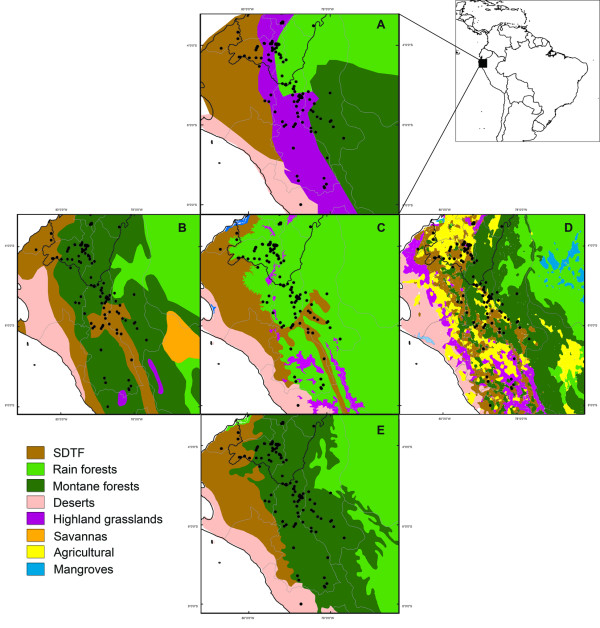
**Performance of the biome maps for the SDTF biome**. Figure illustrating how biome maps differ in depicting biomes at local and regional level. The same area from Northern Peru is shown for each of the five biome maps. The Marañón inter-Andean valley runs through the area in roughly north-south orientation, and is most clearly visible in map B as a brown club-shaped area. The map D depicts a more realistic picture of the river valley, however, showing a narrower valley with an extension of the dry forests further north. A. Morrone's Biogeographic map [30]; B. Americas Base Map [29]; C. WWF Ecoregions [16]; D. Land Cover Map [17]; E. Ecological Systems Map [31].

Regional level comparison of the biome maps shows that the limiting spatial resolution of the biome maps is largely responsible for the poor performance of the SDTF ground-truthing (Figure 2). The Marañón Valley in Northern Peru is one of the most diverse SDTF nuclei in South America, and is geographically easily defined as it is a narrow inter-Andean valley situated between the Western and Eastern Cordilleras. Only three of the maps depict the Marañón Valley (LCM, ECO & AB; Figure 2), whilst two of the maps miss the forest nucleus and categorise the diverse Andean biomes under a single unit (LAB & ESM; Figure 2).

Despite its high spatial resolution the LCM performed poorly in the map comparison recovering only 14.6% of specimens under SDTF (Table 3). The poor performance is an artefact of the LCM including anthropogenic habitats, however. Nearly half of the specimens fall into agricultural land (48.2%) under categories such as 'Mosaic agriculture and degraded forest' (Additional file 1, Table S1). Although strict comparison of the LCM and the other maps is difficult, the results indicate that large fraction of SDTF in South America is affected by human disturbance and is severely fragmented. If the agricultural areas are considered as SDTF, the LCM map becomes the best performing map with 62.7% of specimens mapping under the correct biome (Table 3). Specimens falling outside SDTF map under Cerrado and the Chaco biome, similar to the other maps (Table 3). This indicates that the poor performance of LCM is not because it misidentifies the biome, but due to the severe human-induced fragmentation of the SDTF biome.

### Biome Distribution Modelling

**Figure 3:**
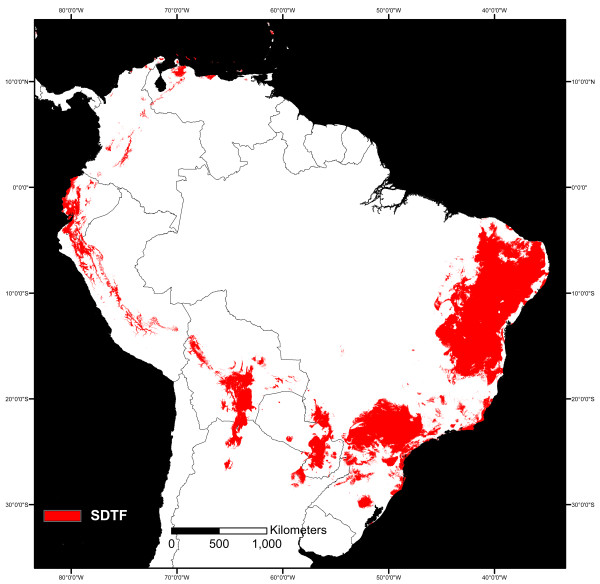
**Modelled distribution of the SDTF biome in South America**. SDTF distribution in South America as predicted by Maxent model using 6,300 herbarium specimens of SDTF habitat specialist species occurrence data and high-resolution (c. 1 km × 1 km) bioclimatic and elevational data for the continent. The logistic output of the model is shown, where areas with high probability of suitable environmental conditions for SDTF are highlighted in red.

For all 10 runs of the occurrence data, resulting training and test AUC values were good (mean AUC = 0.0.832 (SD 0.002) and 0.822 (SD 0.006), respectively). Omission of test and training samples followed closely the predicted omission rate, indicating that the test and training data were independent. Jackknife tests showed that all 20 environmental variables contributed to the model evenly. No variable contained substantial amount of unique information. The environmental variables most important in shaping the model in the training and test data sets as well according to the AUC score were mean temperature of coldest quarter, annual precipitation, precipitation of the wettest month, precipitation of wettest quarter, annual mean temperature, and temperature seasonality. The modelled distribution of SDTF based on one of the 10 runs using 30% of specimens for testing showed highly similar distribution of the biome across South America compared to the most recent schematic map of the biome (Figures 1 and 3). The modelled distribution visualised small SDTF forest nuclei along the Andes, as well as some within the Chaco and the Cerrado biomes (Figure 3).

## Discussion

### Comparison of Current Biome Maps

Baseline data of the current biome maps of South America reviewed here varies considerably, including data on species composition, endemism, climate, elevation, and vegetation structure. Hence, biome delimitations are expected to vary between the maps. The expectation reflects the fact that biomes are complex empirical realities that are hard to organise into fixed categories, an issue discussed in depth in previous publications (e.g. [31,47]). Despite this, there is a growing need to review how biomes are defined in biology [48]. Macroecological and evolutionary research is developing into fields investigating ecological and evolutionary aspects of biomes, such as productivity gradients [21], extinction risk [22], and forest die-back due to climate change across biomes [24]. Such studies should be based on biomes defined as biologically meaningful units, i.e. large evolutionary meta-communities that are not only ecological similar but share evolutionary lineages (species, genera, families, and orders). Ways of deriving such evolutionary biome delimitations using community phylogenetics have been explored in a recent study [Oliveira-Filho AT, Pennington RT, Rotella J, Lavin M: Exploring evolutionarily meaningful vegetation definitions in the tropics: a community phylogenetic approach, submitted].

With this in mind, we performed a detailed comparison of the five biome maps using the SDTF biome as an example. SDTF is a poorly known biome with a strongly fragmented distribution across South America, and hence, it works as a perfect case study for exploring issues in biome mapping. SDTF has been confused in the past with other South American dry biomes, the Chaco and savannas, especially the Brazilian Cerrado and hence we expected to see major differences between maps. We used georeferenced specimen data of SDTF habitat specialist species to ground-truth the biome maps and to test how the maps differed in depicting SDTF distribution.

The results showed poor performance of all maps in depicting known fragments of SDTF based on herbarium records of habitat specialist species. Less than half of specimens were mapped under the SDTF biome in all of the maps. Large proportions of specimens were mapped under other biomes, mainly the Chaco and Cerrado, or under neighbouring biomes in the Andes. Our first step was to fully explore the potential underlying causes of the poor performance. The mismatch between the species distribution data and the biome maps raised the question of whether georeferenced herbarium specimens can be validly used as surrogates for biome distribution. Here we consider two important questions in relation to herbarium data: (1) georeferencing errors, and (2) species' ecological lability and habitat preferences.

Georeferencing errors are common in databases such as GBIF, and rigorous cleaning is required before any analysis can be done (see Methods). Most of the modern herbarium specimens do not present an issue, as these have been georeferenced in the field with GPS and have relatively accurate coordinate data. Excluding obvious typing errors, these modern collections can be considered as high quality data. Specimens without coordinate data, however, are being georeferenced after the actual collection event based on the locality description on the specimen label. This is where errors can take place. Whether the georeferencing is done manually or with automated software, both methods come with errors. The beauty of herbarium data is, however, that each specimen has duplicates, commonly as many as five, which are deposited in other herbaria. As these specimens become georeferenced, they provide independent, repeated samples which can be used to detect errors. Hence we consider the role of georeferencing errors in relation to herbarium data in general as a manageable source of error that can be controlled with rigorous cleaning. In our dataset, duplicate georeferenced specimens allowed efficient cleaning of our datasets, with c. 390 records deleted as a result.

The role of potential "weedy" species (species with a broader ecological preference that spans the STDF limits) was investigated through re-analysing the maps using smaller data sets of specimen records from narrowly restricted endemics only. The narrowly restricted endemics occur in a single or a small set of SDTF nuclei only, rather than across the biome, and can hence be considered as strict habitat specialists. The results from the second analysis supported the wider analysis, indicating that the choice of species did not affect our results.

Excluding the possibility of large georeferencing errors and weedy species, our data from the ground-truthing analysis indicates two major issues with the current biome maps. First we consider the effect of poor spatial resolution. All maps, with the exception of the LCM, showed a breakdown of resolution at regional scale. Such poor spatial resolution strongly limits the use of such maps in GIS applications. This is particularly the case for areas with high elevational heterogeneity where the landscape is naturally fragmented. Our example of the Marañón Valley in Northern Peruvian Andes illustrates how the current maps oversimplify the complex landscape, mainly due to their poor resolution. The only map in our analysis which succeeded in depicting the heterogenous landscape showing smaller SDTF forest nuclei in the Andes was the LCM, a map based on remote-sensed data.

Secondly, we consider the role of poor delimitation of biomes in the current maps. Whilst the LCM does not seem to suffer from lack of spatial resolution at regional scale, it suffers greatly from poor delimitation of the SDTF biome. Small fragments of SDTF depicted in the Andes are labelled under categories such as 'Shrub savannah' and 'Montane forests'. This is not surprising considering how difficult it is to distinguish between dry vegetation types with remote sensed data alone [49]. The poor delimitation of the SDTF and other dry biomes in the LCM suggests that there is a particular need to use ground-truthing or other validation methods in remote sensing, especially for dry biomes.

### Refining SDTF Distribution

So what is the best current estimate of the SDTF distribution? We used the BDM approach to generate a refined distribution map of SDTF, where climatic and elevation data was used in conjunction with the herbarium specimen data to model the biome distribution. The modelled SDTF map strongly agrees with previously published maps [33,45] but is higher in spatial detail. Whilst the current South American biome maps failed in accurately depicting small SDTF fragments such as the Andean forest nuclei, the modelled distribution gives a more realistic representation of the biome in South America. The model performance was good, close to excellent, which gives support to the idea that modelling ecologically similar species under a single model might be a justifiable approach. Previous study of the North American mouse species *Peromyscus polionotus *and its 15 subspecies concluded that modelling ecologically coherent units (i.e. subspecies in their case) resulted in better distribution models compared to models where ecologically divergent subspecies were combined into a single data set [50]. Similar studies should be done to explore model performance when mapping multiple species using the BDM approach.

The availability of a more accurate distribution map for the South American SDTF will hopefully highlight the importance and diversity of the ecosystem, and is a prerequisite for conservation planning and management. For example, despite the small size of the Andean SDTF fragments, depicting their distribution is of great importance, as these forest nuclei host unique biota comparable to the diversity found in the Galápagos Islands (e.g. Marañón Valley, Northern Peru [46,51]). Furthermore, our ground-truthing analysis of the LCM showed that large percentage of SDTF areas are highly degraded due to agriculture. Given that 54.2% of the remaining SDTF are estimated to be in South America based on the recent global overview of the SDTF conservation status [52], our results paint a dire picture of the status of these forgotten forests where approximately half of the forest area has been degraded by agriculture. The remaining areas are becoming smaller and smaller, and hence harder to detect and depict in large scale maps.

### Use of Herbarium Specimen Data in Biome Mapping

What can we learn from this case study? Our analysis shows just how difficult it is to map highly discontinuous and fragmented vegetation like SDTF over large spatial scales. Fragmented biomes are underrepresented in biome maps in general, as smaller fragments are easily missed especially if spatial resolution is poor. Anthropogenic fragmentation poses additional challenges: vegetation cover is becoming increasingly fragmented due to human disturbance, and habitat degradation is leading to changes in vegetation structure even in biomes previously deemed structurally homogeneous. Both of these factors lead to difficulties of mapping biomes based on vegetation structure data alone (i.e. remote sensing).

This is where herbarium data from habitat specialist species could help, given that plants act as indicators of the environment as a whole. With increasing number of georeferenced specimens available through online databases (e.g. over 1.8 million specimens available for Brazil through CRIASpecies link alone), we argue that specimen data can generally contribute to the growing need of feasible validation tools for remote sensing maps (e.g. [53,54]). Whilst ground-truthing over continental scales is not feasible, it can be done *in silico *by downloading and cleaning herbarium data in a relatively short time over large spatial scales. Lack of validation tools has been highlighted in recent reviews as a major area requiring further research [55,56]. Herbarium specimens are currently used in modelling species distributions and in estimating species diversity [57,58], but no studies to our knowledge have explored the use of georeferenced specimen data as a validation tool, despite the availability of millions of specimens available online.

*In silico *ground-truthing would be particularly useful for biome maps of highly environmentally heterogeneous areas such as the Andes. Current continental scale biome maps depict a depauperate picture of Andean diversity concatenating much of it into single meaningless units such as 'Montane vegetation of dry forest and open woodland'. Strongly seasonal biomes, such as SDTF, are another special case where herbarium data can provide help. Remote sensing images are often inadequate in distinguishing seasonal forests, as they can appear like humid forests during wet season, but as shrubland during the long dry season. Highly degraded biomes and habitats provide yet another case where herbarium data could be used to study habitat loss over time, as specimen data over time can provide an estimate of the original distribution of vegetation cover based on plants collected before land clearance. Lastly, human-induced disturbance and habitat degradation causes issues in remote sensing, and herbarium data could be used as an aid in distinguishing between degraded savanna and degraded dry forest which is currently not feasible with remote sensed data alone.

Another use of herbarium data in biome mapping is the BDM approach presented in this paper. The BDM approach has previously been used to map historical distribution of biomes using past climate conditions in combination with herbarium data [59-61], whilst our focus was to use the approach to model current biome distribution. The advantage of the BDM approach over other mapping methods is that it combines high spatial resolution environmental data with floristic data in the form of georeferenced herbarium specimens. The approach results in maps with extremely high spatial resolution (1 km × 1 km) and requires less ground-truthing as maps are modelled based on floristic data. In the case of SDTF, BDM approach produced a much improved biome map with a relatively small effort. The new map can be considered as a working hypothesis of the SDTF distribution in South America, and as more data is added to the model, the distribution of the biome can be easily refined.

## Conclusions

Current biome maps of South America perform poorly in depicting known fragments of SDTF which are based on herbarium records of habitat specialist species. The poor performance of the maps can be attributed to two main factors: (1) the poor spatial resolution of the biome maps, and (2) their poor delimitation of SDTF. Georeferenced herbarium data could provide a validation tool for enhancing biome maps in general. Map schemes that rely fully on remote sensed data could gain from the use of herbarium specimens in particular, as ground-truthing across continents with plot data is currently not feasible. The lack of studies incorporating herbarium specimens has been likely due to inadequate specimen data across species distributions especially for tropical taxa [62], but the situations is rapidly improving as more information is collected and digitized, potentially leading to its use not only in validating biome maps, but also in constructing them [48]. An alternative approach is presented where herbarium specimens are used in conjunction with environmental data to model current biome distributions. Incorporating herbarium data in biome mapping using either of the above approaches should be encouraged, especially so in projects focusing on poorly known, fragmented and/or structurally heterogeneous biomes. We highlight that special attention should be given to specimen identification. Specimen determinations by taxonomic experts should be used as a way to quality control data. Taxonomic sources should also be consulted in the choice of species used.

## Methods

### SDTF Habitat Specialist Species Occurrence Data Set

Georeferenced herbarium specimen data of endemic SDTF habitat specialist species was used to test the accuracy of the available biome maps. Despite the generally high ß-diversity among SDTF nuclei, there are a small set of widespread species that occur in most of the forest nuclei across South American SDTFs [44]. Despite their wide distribution across the continent, these species are considered as habitat specialists, and all of the nine species were included in this study. A set of further 23 species have been used to define the SDTF distribution in previous publications [32,33,45]. Although recent data indicates that many of these species are ecologically more labile than previously thought (e.g. *Anadenanthera colubrina *[63]), we included the 23 species in the data matrix. Lastly, in order to reach a more comprehensive species list, we identified 23 narrowly distributed endemics from different SDTF nuclei (e.g. *Cyathostegia matthewsii, Solanum plowmanii*, Table 5). These narrowly distributed species acted also to boost specimen numbers for generally poorly collected areas such as Andean Peru and Bolivia. The final list included a total of 49 species (Table 5). Occurrence data for the selected species were downloaded from GBIF Data Portal (data.gbif.org, August 2011), CRIA speciesLink (splink.cria.org.br, August 2011), and the Solanaceae source (http://www.nhm.ac.uk/research-curation/research/projects/solanaceaesource/, August 2011).

**Table 5:** List of SDTF specialist species

**No**.	Species	Family	**Prado & Gibbs **[32]	**Prado **[33]	**Linares-Palomino et al**. [44]	No. of specimens included
**1**	*Amburana cearensis *(Fr.All.) A.C.Smith	Leguminosae	x	x	SDTF generalist	243
**2**	*Anadenanthera colubrina *(Vell.) Brenan	Leguminosae	x	x	**x**	714
**3**	*Aspidosperma polyneuron *Müll. Arg.	Apocynaceae	x		x	171
**4**	*Aspidosperma pyrifolium *Mart.	Apocynaceae	x	x	**x**	300
**5**	*Balfourodendron riedelianum *(Engl.) Engl.	Rutaceae		x	x	53
**6**	*Blanchetiodendron blanchetii *(Benth.) Barneby & J.W. Grimes	Leguminosae				30
**7**	*Chloroleucon tenuiflorum *(Benth.) Barneby & J.W. Grimes	Leguminosae			x	47
**8**	*Combretum leprosum *Mart. Search in The Plant List	Combretaceae	x	x	x	78
**9**	*Cordia americana *(L.) Gottschling & J.S. Mill.	Boraginaceae	x	x	x	103
**10**	*Cordia incognita *Gottschling & J.S. Mill.	Boraginaceae	x	x		57
**11**	*Cyathostegia matthewsii *(Benth.) Schery	Leguminosae			x	78
**12**	*Diatenopteryx sorbifolia *Radlk.	Sapindaceae	x	x	x	96
**13**	*Enterolobium contortisiliquum *(Vell.) Morong	Leguminosae	x	x	x	304
**14**	*Geoffroea spinosa *Jacq.	Leguminosae	x		x	110
**15**	*Machaerium aculeatum *Raddi	Leguminosae				174
**16**	*Machaerium condensatum *Kuhlm. & Hoehne	Leguminosae			x	20
**17**	*Machaerium ruddianum *Mendonça Filho & A. M. G. Azevedo	Leguminosae				13
**18**	*Mimosa arenosa *(Willd.) Poir.	Leguminosae	x		x	149
**19**	*Myracrodruon urundeuva *Fr.All.	Anacardiaceae	x	x	**x**	452
**20**	*Nicotiana glutinosa *L.	Solanaceae				15
**21**	*Parapiptadenia blanchetii *(Benth.) Vaz & Lima	Leguminosae				23
**22**	*Parapiptadenia zehntneri *(Harms) M. P. M. de Lima & H. C. de Lima	Leguminosae				125
**23**	*Peltogyne pauciflora *Benth.	Leguminosae				75
**24**	*Peltophorum dubium *(Spreng.) Taub.	Leguminosae	x	x	x	216
**25**	*Phytolacca dioica *L.	Phytolaccaceae	x	x	x	216
**26**	*Piptadenia viridiflora *(Kunth) Benth.	Leguminosae	x	x		216
**27**	*Pityrocarpa moniliformis *(Benth.) Luckow & R. W. Jobson	Leguminosae				188
**28**	*Pouteria gardneriana *(A. DC.) Radlk. Search in The Plant List	Sapotaceae	x	x	x	64
**29**	*Pterogyne nitens *Tul.	Leguminosae	x	x	SDTF generalist	323
**30**	*Ruprechtia laxiflora *Meissn.	Polygonaceae	x	x	x	266
**31**	*Schinopsis brasiliensis *Engl.	Anacardiaceae	x	x	x	286
**32**	*Sideroxylon obtusifolium *(Roem. & Schult.) T.D.Penn.	Sapotaceae	x		**x**	317
**33**	*Solanum amotapense *Svenson	Solanaceae				13
**34**	*Solanum chmielewskii *(C.M.Rick et al.) D.M.Spooner et al.	Solanaceae				7
**35**	*Solanum confertiseriatum *Bitter	Solanaceae			x	70
**36**	*Solanum corumbense *S.Moore	Solanaceae				35
**37**	*Solanum daphnophyllum *Bitter	Solanaceae				55
**38**	*Solanum gnaphalocarpon *Vell.	Solanaceae			x	7
**39**	*Solanum granuloso-leprosum *Dunal	Solanaceae	x		x	36
**40**	*Solanum hibernum *Bohs	Solanaceae				14
**41**	*Solanum huaylasense *Peralta,	Solanaceae				1
**42**	*Solanum hutchisonii *(J.F.Macbr.) Bohs	Solanaceae				4
**43**	*Solanum iltisii *K.E.Roe	Solanaceae			x	20
**44**	*Solanum neorickii *D.M.Spooner et al.	Solanaceae				24
**45**	*Solanum plowmanii *S.Knapp	Solanaceae			x	27
**46**	*Solanum smithii *S.Knapp	Solanaceae				15
**47**	*Solanum stuckertii *Bitter	Solanaceae				19
**48**	*Ximenia americana *L.	Ximeniaceae	x		**x**	232
**49**	*Zanthoxylum fagara *(L.) Sarg.	Rutaceae			**x**	199
					TOTAL	**6300**

List of species used for ground-truthing the biome maps. See Methods for details on how species were chosen. Species highlighted in grey are narrowly distributed endemics restricted to only a few of the SDTF nuclei. These were used in a secondary analysis where the effect of widespread and potentially weedy species was investigated. Species used as SDTF indicators in previous studies are shown (x). Widespread but ecological specialised SDTF species defined by Linares-Palomino et al. [44] are shown in bold (**x**).

**Figure 4:**
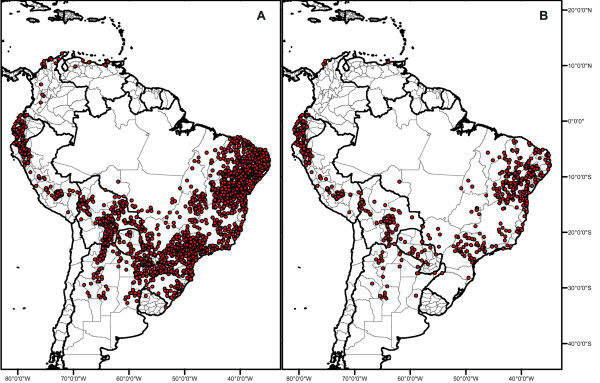
**Distribution map of the SDTF specialist species**. Combined distribution map of the herbarium records used in this study. A. Map based on 6,300 specimen records of 49 SDTF specialist species; B. Map based on a reduced dataset of 1,110 specimen records where 23 narrowly distributed species with more restricted ecological ranges were included.

The data set was cleaned by comparing distributions to areas noted in original taxonomic publications. Obvious outliers were checked and deleted when necessary. Duplicate specimens were used to check the data quality; when duplicates with different coordinates were spotted, these were deleted (390 specimens in total). The resolution of the georeferenced material varied from poor (degrees only) to excellent (degrees, minutes and seconds). Specimens with degrees only were excluded from the datasets. Cultivated specimens were excluded. The final cleaned datasets included a total of 6,300 specimens, and 3,733 unique localities from across the SDTF biome distribution (Figure 4). Secondary analysis was done with a smaller data set (1,110 specimens) to test the effect of the widely distributed species in our analysis (Table 5; Figure 4). The secondary analysis included the 23 narrowly distributed endemic species only (Table 5). Most of the widely distributed species were discarded from this analysis due to recent data indicating doubts on their habitat preferences [63].

The combined distribution map of the specimens, both the full and partial dataset (Figure 4) was drawn using ArcMap 10, and was observed to match the SDTF distribution depicted in previous publications [33,45]. Although no obvious gaps in the distribution data can be identified, the dataset had only a few specimens from northern South America (Figure 4). Most data points were for Brazil, Argentina, Paraguay and Bolivia.

### Comparison of Biome Maps

The maps included in the study are freely available online or can be requested from the corresponding authors. Attribute tables of biome maps were used to obtain data on their hierarchical divisions using ArcMap 10. The number of divisions was recorded for South America only, as this was the largest common denominator of all the maps. The Caribbean and the islands of the coast of South America were excluded. Urban and barren areas (e.g. water, ice, and snow) were omitted from each biome map prior to calculations.

Map comparison was performed in ArcMap 10 using the full and partial herbarium specimen data sets as a way of ground truthing. The ArcToolbox option of Spatial Join was used to join the distribution data with the biome map layer. Once the joined data file was created, the number of specimens falling into each biome category was calculated using the enquiry tool. Specimens that fell under categories 'Shrublands' and 'Deserts and xeric shrublands' were included under SDTF (Additional file 1). Because maps LAB and ESM did not distinguish SDTF under a single category, areas 'Caatinga', 'Arid Ecuador', 'Tumbes-Piura', and 'Monte' in LAB, and 'Caatinga', 'Dry Meso-America', and 'Caribbean' in ESM, were regarded as SDTF (Additional file 1).

### Distribution Modelling of SDTF

A new map for the SDTF was constructed using an approach here referred to as the biome distribution modelling (BDM). BDM is based on species distribution modelling, where environmental variables are used in conjunction with species occurrence data to model species distributions. Instead of modelling a single species distribution, BDM uses a composite data set of habitat specialist species to model the distribution of the whole biome.

BDM was performed using the maximum entropy model as implemented in Maxent software [64,65] as the model has been shown to perform well against other presence only models [66]. The model uses the principle of maximum entropy density estimation to generate a probability distribution based on presence-only data [64,65]. A single model was constructed for the South America SDTF using the complete herbarium specimen data set with 6,300 records. Input environmental variables included 19 bioclimatic variables and elevation data from WorldClim at 30 arc-second spatial resolution (c. 1 km^2^, http://www.worldclim.org/bioclim) [67]. The layers were downloaded in tiles, including tiles 23-24, 33-34, and 43-44. The entire set of 19 climatic variables was used to avoid any a priori assumptions of correlations among the variables. Maxent 3.3.2 (http://www.cs.princeton.edu/) was run with default settings: convergence threshold 10^-5^, maximum number of iterations of 500, regularisation = 1. Distribution data set was partitioned so that 30% of the records were omitted from model building and used as a test dataset (1,025 specimens). Ten iterations of the model were run with random seed to derive mean and standard deviation (SD) of AUC model scores. The model output was evaluated using the area under curve (AUC) value of receiver operating characteristic (ROC) plot. AUC value of 1 indicates optimal performance, whilst AUC = 0.5 indicates performance equal to random. The importance of the input environmental variables in model building was measured using jackknife. Jackknife test compares gains between models run with and without each environmental variable and measures the relative importance of each variable to the final model build. The resulting distribution is given in logistical values, where 0 refers to low probability and values near 1 mean high probability of presence. Map was generated by visualising all areas with logistical value > 0.5. Omission levels at this level were 36% for training and 37% for testing data set. The map is available from the authors by request.

## Authors' contributions

TS led the design of the study with considerable contributions from all authors. TS analysed the data and drafted the manuscript, and TS and JRVI assembled and cleaned the data set. DP led the choice of specialist species and the overall concept of SDTF in biome maps as the senior leading researcher. All authors assisted in writing the manuscript, and read and approved the final manuscript.

## Supplementary Material

Additional file 1 **Tables S1-S6**. Results of each of the biome maps and their performance using specimen data. Biomes corresponding to SDTF are highlighted for each map (file available electronically). Click here for file

Additional file 2 **Tables S7-S8**. Species-by-species breakdown of results for the WWF Ecoregion and the Land Cover Map (file available electronically). Click here for file
