# Supplementary material for: Forgotten forests - issues and prospects in biome mapping using Seasonally Dry Tropical Forests as a case study
Source: BMC Ecol. 2011 Nov 24;11:27. doi: 10.1186/1472-6785-11-27 (PMC3254131; doi:10.1186/1472-6785-11-27)
Supplement: Additional file 1 — Tables S1-S6. Results of each of the biome maps and their performance using specimen data. Biomes corresponding to SDTF are highlighted for each map (file available electronically). [file 1472-6785-11-27-S1.DOC]

# Additional file 1 – Results of the individual map analysis

Särkinen et al. “Forgotten forests of South America – comparing biome delimitations between land use and biome maps in relation to dry vegetation types”

### Contents

Table S1. Results of the Land Cover Map by Eva et al. [17].

Table S2. Results of the WWF Ecoregion map [16].

Table S3. Results of the Americas Base Map [29].

Table S4. Results of the map by Morrone [30].

Table S5. Results of the Ecological Systems Map [31].

Table S6. Areas labelled as non-SDTF but which received more than 20 specimen hits in our analysis.

Table S1. Results of the Land Use Map by Eva et al. [17]. Biomes considered as part of the SDTF biome are highlighted in grey.

| **Biome** | All specimen data | | Narrow endemics only | |
| --- | --- | --- | --- | --- |
|  | **No. of specimens** | **Percentage of total** | **No. of specimens** | **Percentage of total** |
| Agricultural | 3035 | 48.2 | 453 | 41.1 |
| Montane forests | 725 | 11.5 | 216 | 19.6 |
| Barren (urban + ice & rock) | 684 | 10.9 | 92 | 8.4 |
| Shrublands | 640 | 10.2 | 158 | 14.4 |
| Savannas | 431 | 6.8 | 53 | 4.8 |
| SDTF | 278 | 4.4 | 20 | 1.8 |
| Rain forests | 254 | 4.0 | 34 | 3.1 |
| Chaco | 139 | 2.2 | 24 | 2.2 |
| Temperate grasslands | 64 | 1.0 | 14 | 1.3 |
| Montane grasslands | 46 | 0.7 | 34 | 3.1 |
| Mangroves | 3 | < 0.0 | 2 | 0.2 |

Table S2. Results of the WWF Ecoregion map [16]. Biomes considered as part of the SDTF biome are highlighted in grey.

| # | **Biomes** | All specimen data | | Narrow endemics only | |
| --- | --- | --- | --- | --- | --- |
|  |  | **No. of specimens** | **Percentage of total** | **No. of specimens** | **Percentage of total** |
| 13 | Deserts and xeric shrublands | 2,271 | 36.0 | 335 | 30.4 |
| 1 | Tropical and subtropical moist broadleaf forests | 2,124 | 33.7 | 463 | 42.1 |
| 7 | Tropical and subtropical grasslands, savannas and shrublands | 1,065 | 16.9 | 117 | 10.6 |
| 2 | Tropical and subtropical dry broadleaf forests | 664 | 10.5 | 149 | 13.5 |
| 9 | Flooded grasslands and savannas | 66 | 1.0 | 3 | 0.3 |
| 14 | Mangroves | 39 | 0.6 | 6 | 0.5 |
| 0 | Ice, water, rock | 29 | 0.5 | 11 | 1.0 |
| 10 | Montane grasslands and shrublands | 22 | 0.3 | 15 | 1.4 |
| 8 | Temperate grasslands, savannas and shrublands | 19 | 0.3 | 2 | 0.2 |

Table S3. Results of the Americas Base Map [29]. Biomes considered as part of the SDTF biome are highlighted in grey. Despite reference to dry forets, biome 35 is not considered part of the SDTF biome but refers to a mix of montane forests and tropical highland grasslands.

| **#** | **Biome** | All specimen data | | Narrow endemics only | |
| --- | --- | --- | --- | --- | --- |
|  |  | **No. of specimens** | **Percentage of total** | **No. of specimens** | **Percentage of total** |
| 34 | Dry, partly sclerophyllous forest and open woodland | 2,690 | 42.7 | 462 | 42.0 |
| 32 | Seasonally wet forest, wet savanna | 1,883 | 29.9 | 170 | 15.4 |
| 42 | Floodplain tropical vegetation | 535 | 8.5 | 87 | 7.9 |
| 45 | Mountain vegetation of rain forest | 316 | 5.0 | 154 | 14.0 |
| 35 | Mountain vegetation of dry forest and open woodland | 209 | 3.3 | 107 | 9.7 |
| 43 | Seaside littoral vegetation | 163 | 2.6 | 35 | 3.2 |
| 31 | Mountain vegetation of monsoon rain forest | 137 | 2.2 | 20 | 0.5 |
| 46 | Evergreen and partly deciduous forests, open woodland and shrubland | 139 | 2.2 | 6 | 1.8 |
| 44 | Rain forest (Amazon basin & Choco) | 76 | 1.2 | 22 | 2.0 |
| 30 | Rain forest (Mata Atlantica) | 70 | 1.1 | 14 | 1.3 |
| 48 | Grassland | 31 | 0.5 | 1 | 0.1 |
| 0 | Barren land | 22 | 0.3 | 5 | 0.5 |
| 40 | Mountain vegetation of desert and semidesert | 12 | 0.2 | 11 | 0.4 |
| 33 | Mountain vegetation of seasonally wet forest and savanna | 11 | 0.2 | 4 | 0.3 |
| 39 | Desert (Pacific coast of Peru & Chile) | 4 | 0.1 | 3 | 1.0 |
| 51 | Desert (temperate & subtropical, in Eastern Cordillera in Argentina) | 1 | < 0.0 | - | - |

Table S4. Results of the map by Morrone [30]. Biomes considered as part of the SDTF biome are highlighted in grey.

| **#** | **Biome** | All specimen data | | Narrow endemics only | |
| --- | --- | --- | --- | --- | --- |
|  |  | **No. of specimens** | **Percentage of total** | **No. of specimens** | **Percentage of total** |
| 48 | Caatinga | 2,514 | 39.9 | 361 | 32.8 |
| 54 | Parana Forest | 948 | 15.0 | 97 | 8.8 |
| 50 | Chaco | 529 | 8.4 | 63 | 5.7 |
| 49 | Cerrado | 476 | 7.6 | 47 | 4.3 |
| 53 | Brazilian Atlantic Forest | 452 | 7.2 | 70 | 6.4 |
| 47 | Yungas | 380 | 6.0 | 124 | 11.3 |
| 55 | Araucaria angustifolia For | 233 | 3.7 | 7 | 0.6 |
| 46 | Pantanal | 202 | 3.2 | 13 | 1.2 |
| 58 | Puna | 164 | 2.6 | 114 | 10.4 |
| 0 | Barren/coastal | 92 | 1.5 | 30 | 2.7 |
| 51 | Pampa | 77 | 1.2 | 1 | 0.1 |
| 35 | Napo | 50 | 0.8 | 40 | 3.6 |
| 30 | Cauca | 39 | 0.6 | 32 | 2.9 |
| 56 | North Andean Paramo | 37 | 0.6 | 32 | 2.9 |
| 32 | Western Ecuador | 28 | 0.4 | 27 | 2.5 |
| 33 | Arid Ecuador | 24 | 0.4 | 23 | 2.1 |
| 34 | Tumbes-Piura | 16 | 0.3 | 15 | 1.4 |
| 25 | Maracaibo | 13 | 0.2 | 1 | 0.1 |
| 26 | Venezuelan Coast | 8 | 0.1 | 1 | 0.1 |
| 45 | Para | 7 | 0.1 | - | - |
| 52 | Monte | 5 | 0.1 | 1 | 0.1 |
| 47 | Ucayali | 3 | 0.0 | - | - |
| 57 | Costal Peruvian Desert | 2 | 0.0 | 2 | 0.2 |

Table S5. Results of the Ecological Systems Map [31]. Biomes considered as part of the SDTF biome are highlighted in grey.

|  |  | All specimen data | | Narrow endemics only | |
| --- | --- | --- | --- | --- | --- |
| **#** | **Division** | **No. of species** | **Percentage of total** | **No. of species** | **Percentage of total** |
| 501 | Caatinga | 2,263 | 35.9 | 329 | 29.9 |
| 410 | Atlantic Forest | 1,846 | 29.3 | 215 | 19.5 |
| 406 | Cerrado | 876 | 13.9 | 73 | 6.6 |
| 409 | North-Central Moist Andes | 630 | 10.0 | 317 | 28.8 |
| 502 | Chaco | 420 | 6.7 | 61 | 5.5 |
| 602 | Pampas | 76 | 1.2 | 1 | 0.1 |
| 402 | Moist Meso-America | 55 | 0.9 | 48 | 4.4 |
| 408 | Amazonia | 52 | 0.8 | 11 | 1.0 |
| 401 | Dry Meso-America | 26 | 0.4 | 25 | 2.3 |
| 0 | Empty/barren land | 26 | 0.4 | 10 | 0.9 |
| 411 | Caribbean | 16 | 0.3 | 3 | 0.3 |
| 505 | South-Central Dry Andes | 6 | 0.1 | 5 | 0.5 |
| 504 | Peruvian-Chilean Desert | 3 | < 0.0 | 3 | 0.3 |
| 604 | Patagonia | 3 | < 0.0 | - | - |
| 405 | Orinoquia | 1 | < 0.0 | - | - |
| 406 | Cerrado | 1 | < 0.0 | - | - |

Table S1. Areas labelled as non-SDTF but which received more than 20 specimen hits in our analysis. Results for the Land Cover Map (LCM; [17]), WWF Ecoregions (ECO; [16]), and the Americas Basemap (AB; [29]) are shown.

| **Biome map** | **No. of specimen hits** | **Grid ID number** | **Biome** | **Details of area from original biome maps** |
| --- | --- | --- | --- | --- |
| AB | 1220 | 276 | Savannas | Southern Brazil: South Brazilian (Cedrela fissilis, C. glaziovii, Hymenaeastilbocarpa, Aspidosperma polyneuron, Inga edulis) |
| AB | 380 | 209 | Floodplain tropical vegetation | Northeast Brazilian (Hymenaea courbaril, species of gg. Bowdichia, Erythrina) |
| AB | 322 | 186 | Savannas | Brazilian--(Locally, "campos--cerrados," woodland savanna.)(Curatella americana, species of gg. Kielmeyera, Byrsonima, Erythroxylum, Dimorphandra; grasses: speciesof gg. Paspalum, Panicum, Andropogon, Aristida) |
| AB | 138 | 249 | Mountain vegetation of rain forests | Bolivian (species of gg. Gaultheria, Podocarpus, Cyathea) |
| AB | 136 | 326 | Evegreen and partly deciduous forsts, open woodland and shrubland | South Brazilian (Araucaria angustifolia, species of g. Podocarpus, Cedrela fissilis, Ilex paraguayensis, Phoebe porosa, Dicksonia sellowiana) |
| AB | 85 | 94 | Mountain vegetation of rain forests | Equadorian--Peruvian (Podocarpus nubigenus, Oreopanax artocaroides, Clusia pseudomangle) |
| AB | 84 | 269 | Savannas | Pantanal (Tecoma caraiba, Ceiba dlaziovii, Parantheria prostrata; grasses: species of gg. Paspalum, Panicum, Oryza) with deciduous forest |
| AB | 79 | 171 | Seaside littoral vegetation | Brazilian (species of gg. Iresine, Ipomaea, Hydrocotyle, Leucothoe, Gaylussacia, Cereus, Melocactus) |
| AB | 73 | 319 | Mountain vegetation of monsoon rain forest | West Atlantic (Piptadenia rigida, P. communis, Caesalpinia echinata, Dalbergia nigra, Tecoma heptaphylla, Nectandra grandiflora, Phoebe porosa, Ceiba pentandra) |
| AB | 69 | 263 | Savannas | Bolivian (Astronium urundeuva, Piptadenia macrocarpa, Hymenaea stilbocarpa) |
| AB | 65 | 194 | Mountain vegetation of monsoon rain forest | West Atlantic (Piptadenia rigida, P. communis, Caesalpinia echinata, Dalbergia nigra, Tecoma heptaphylla, Nectandra grandiflora, Phoebe porosa, Ceiba pentandra) |
| AB | 49 | 189 | Rain forest (Amazon basin & Choco) | Southwest Amazonian (Swietenia macrophylla, Myroxylon balsamum, Torresea acreana, Calophyllum brasiliense, species of gg. Cedrela, Virola) |
| AB | 47 | 257 | Rain forest (Mata Atlantica) | West Atlantic (Piptadenia rigida, P. communis, Hymenaea stilbocarpa, Melanoxylon brauna, Centrolobium robustum, Dalbergia nigra) |
| AB | 45 | 255 | Seaside littoral vegetation | Brazilian (species of gg. Iresine, Ipomaea, Hydrocotyle, Leucothoe, Gaylussacia, Cereus, Melocactus) |
| AB | 41 | 342 | Savannas | Argentine (Astronium balansae, Tabebuia ipe, Luehea divaricata, Peltophorum dubium, Piptadenia rigida, Cedrela fissitis) |
| AB | 40 | 348 | Floodplain tropical vegetation | Argentine (Salix humboldtiana, Ficus monckii, Phytolacca dioica, Ocotea acutifolia) |
| AB | 38 | 287 | Savannas | Bolivian (species of gg. Tecoma, Bombax, Ceiba, Chlorophora, Aspidosperma; grasses: species of gg. Tristachya, Andropogon, Paspalum) with palm savanna |
| AB | 30 | 162 | Floodplain tropical vegetation | Northeast Brazilian (Hymenaea courbaril, species of gg. Bowdichia, Erythrina) |
| AB | 28 | 358 | Savannas | Uruguayan (Generium argenteum, Paspalum notatum, Axonopus compressus, Sporobolus poiretii, Andropogon saccharoides, Eragrostis neesii) with shrubland (Erythrina cristagalli, Prosopis algarobilla, species of gg. Schinus, Acacia, Colletia) |
| AB | 26 | 300 | Mountain vegetation of rain forests | Selva--Yungas (species of gg. Hufelandia, Endlicheria, Nectandra--species of gg. Ocotea, Phoebe, Cinechona) |
| AB | 21 | 175 | Mountain vegetation of rain forests | Selva--Yungas (species of gg. Hufelandia, Endlicheria, Nectandra--species of gg. Ocotea, Phoebe, Cinechona) |
| AB | 21 | 361 | Savannas | South Brazilian (Cedrela fissilis, C. glaziovii, Hymenaeastilbocarpa, Aspidosperma polyneuron, Inga edulis) |
| ECO | 719 | 60150 | Tropical and subtropical moist broadleaf forest | Alto Paraná Atlantic forests |
| ECO | 623 | 60704 | Savanna | Cerrado |
| ECO | 252 | 60104 | Tropical and subtropical moist broadleaf forest | Bahia interior forests |
| ECO | 219 | 60160 | Tropical and subtropical moist broadleaf forest | Serra do Mar coastal forests |
| ECO | 206 | 60708 | Chaco | Humid Chaco |
| ECO | 169 | 60105 | Tropical and subtropical moist broadleaf forest | Bolivian Yungas |
| ECO | 161 | 60210 | Chaco | Dry Chaco |
| ECO | 159 | 60101 | Tropical and subtropical moist broadleaf forest | Araucaria moist forests |
| ECO | 134 | 60121 | Tropical and subtropical moist broadleaf forest | Eastern Cordillera real montane forests |
| ECO | 108 | 60103 | Tropical and subtropical moist broadleaf forest | Bahia coastal forests |
| ECO | 98 | 60165 | Tropical and subtropical moist broadleaf forest | Southern Andean Yungas |
| ECO | 86 | 60153 | Tropical and subtropical moist broadleaf forest | Peruvian Yungas |
| ECO | 49 | 60152 | Tropical and subtropical moist broadleaf forest | Pernambuco interior forests |
| ECO | 48 | 60710 | Savannas | Uruguayan savanna |
| ECO | 44 | 60907 | Savannas | Pantanal |
| ECO | 41 | 60178 | Tropical and subtropical moist broadleaf forest | Western Ecuador moist forests |
| ECO | 40 | 61406 | Mangroves | Southern Atlantic mangroves |
| ECO | 29 | 60151 | Tropical and subtropical moist broadleaf forest | Pernambuco coastal forests |
| ECO | 28 | 60703 | Savannas | Campos Rupestres montane savanna |
| LCM | 81 | 45427 | Savannas | Shrub savannah |
| LCM | 78 | 38218 | Montane forests | Montane forest >1000m closed deciduous |
| LCM | 56 | 41550 | Savannas | Shrub savannah |
| LCM | 46 | 31531 | Savannas | Shrub savannah |
| LCM | 34 | 16583 | Montane forests | Montane forest 500-1000m open deciduous |
| LCM | 28 | 11596 | Tropical and subtropical moist broadleaf forest | Open semi-humid forest |
